# Biotechnological Tools for the Production of Low-FODMAP Wholegrain Wheat and Rye Cookies and Crackers

**DOI:** 10.3390/foods14040582

**Published:** 2025-02-10

**Authors:** Aleksandra M. Torbica, Bojana Filipčev, Vesna Vujasinović, Uroš Miljić, Goran Radivojević, Milorad Miljić, Miloš Radosavljević

**Affiliations:** 1 Institute of Food Technology, University of Novi Sad, Bulevar Cara Lazara 1, 21102 Novi Sad, Serbia; bojana.filipcev@fins.uns.ac.rs (B.F.); milorad.miljic@fins.uns.ac.rs (M.M.); 2 Faculty of Sciences, University of Novi Sad, Trg Dositeja Obradovića 3, 21000 Novi Sad, Serbia; vesna.vujasinovic@dgt.uns.ac.rs (V.V.); goran.radivojevic@dgt.uns.ac.rs (G.R.); 3 Faculty of Technology, University of Novi Sad, Bulevar Cara Lazara 1, 21102 Novi Sad, Serbia; urosch@uns.ac.rs (U.M.); milosr@tf.uns.ac.rs (M.R.)

**Keywords:** fructooligosaccharides, galactooligosaccharides, wholegrain flours, FODMAP, baker’s yeast, confectionary products, fermentation, malting, herbal extract

## Abstract

Fermentable oligosaccharides, di- and monosaccharides, and polyols defined as FODMAPs readily trigger the symptoms of irritable bowel syndrome (IBS), which affects up to 23% of the population, through several mechanisms. A low-FODMAP diet is a short-term solution due to significant nutrient deficiencies, especially in dietary fibre (DF). IBS patients must avoid cereals, especially wholegrain cereals such as wheat and rye, which are an important natural source of DF and therefore FODMAPs (part of soluble DF). This study is the first of its kind to employ biotechnological tools for the creation of wholegrain low-FODMAP cookies and crackers based on wholegrain wheat and rye flours with high FODMAP contents. Endogenous enzymes activated via prolonged dough resting and exogenously activated enzymes originating from chicory extract, wheat malt, and baker’s yeast were employed. The prolonged dough resting time and the addition of wheat malt reduced the FODMAP content in the wholegrain wheat and rye cookies by 46% and 99.5%, respectively. The best result was achieved in the wholegrain wheat crackers, with a FODMAP content reduction of 59.3% based on the combination of a prolonged dough resting time and the addition of wheat malt and baker’s yeast. In the wholegrain rye crackers, a prolonged resting time alone was sufficient to achieve an 83.6% reduction in the total oligosaccharide content.

## 1. Introduction

The label FODMAP defines fermentable oligosaccharides, di- and monosaccharides, and polyols that are poorly absorbed in the small intestine and thus rapidly fermented in the colon by indigenous microorganisms; they readily trigger the symptoms of irritable bowel syndrome (IBS) [1]. FODMAPs can affect individuals, especially those with IBS or other gastrointestinal disorders, via several mechanisms, leading to symptoms such as bloating, gas, constipation, diarrhoea, and abdominal pain [2,3]. These mechanisms include an increase in gas and water retention and production, leading to luminal distension, the modulation of visceral hypersensitivity, increases in intestinal permeability, the induction of microbiota changes, and the production of short-chain fatty acids (SCFAs), as well as alterations in motility [4]. IBS is the disorder most often disorder diagnosed by gastroenterologists and affects between 9% and 23% of the population [5]. The implementation of a low-FODMAP diet, i.e., a dietary approach restricting the intake of poorly absorbable, fermentable carbohydrates, has become accepted in the treatment of IBS among both gastroenterologists and dieticians because it significantly reduces the symptoms of IBS [6].

These fermentable oligosaccharides include fructans, fructooligosaccharides (FOS), inulin and galactans (mainly galactooligosaccharides (GOS)), raffinose, stachyose, and verbascose. The disaccharides that are classified as FODMAPs include lactose, the monosaccharides include fructose, and the polyols include sorbitol, mannitol, xylitol, and maltitol. According to the most recent evidence, melibiose is also included in the FODMAP category [7,8]. FODMAPs are found in a wide variety of foods, including lactose in milk and yoghurt; fructose in honey and fruits; inulin and FOS in garlic, onion, rye, wheat, banana, and tomato; GOS in legumes; and polyols in stone fruits, mushrooms, and several artificial sweeteners [9]. Wheat and rye are major contributors to the dietary intake of low-molecular-weight fructans, but wholegrain products are also major contributors to the intake of dietary fibre [10].

To comply with the FODMAP recommendations, the cut-off value for each specific FODMAP compound and category is definable and ranges from 0.15 to 1 g per serving/meal for IBS patients. However, diet therapy based on the avoidance of FODMAPs restricts food choices too extensively, eliminating staple foods, likely including wheat derivatives and various vegetables, legumes, and fruits. With this dietary regimen, IBS patients could suffer from a reduced intake of fibre, minerals, vitamins, and phytochemicals. In addition to this unbalanced nutritional status, the drastic reduction in FODMAPs interferes with the intestinal microbiota and colonocyte metabolism [11].

Wheat and wheat-based products only account for one-fourth of the daily calorie intake in the European population, and a significant portion of this comprises bakery products that are designated as staple foods. However, due to the naturally occurring FODMAPs in cereals such as wheat or rye (where fructans and FOS predominate), products made from these grains must be largely avoided by IBS patients [8,12]. Cookies are eaten worldwide, meaning that they are the most consumed confectionary products, and they are suitable for all age groups [13].

Recent research has focused on boosting the nutritional profile of cookies, especially in terms of enriching their dietary fibre content from various sources (mostly the by-products from fruit processing) [14]. An increased fibre content was shown to improve the textural properties of cookies, including their hardness, fracturability, and chewiness [15]. These findings suggest that fortifying low-FODMAP biscuits with high fibre contents could potentially serve as a beneficial intervention for alleviating IBS symptoms [16].

Crackers are considered popular snack products and are in clear demand amongst consumers. Crackers are generally defined as thin, dry, and crisp bakery products, usually made of wheat flour, salt, shortening (or fat), and leavening agents (yeast or chemical rising agents, or a combination of both) [17]. There are only two cracker products labelled as “low-FODMAP” [18]. Although cookies and crackers have similar major ingredients, the main difference, aside from their sugar content, is the application of chemical (cookies) and chemical and/or biological raising agents (crackers).

Taking into the account that the majority of the current research on the creation of low-FODMAP cookies and crackers is based on choosing low-FODMAP ingredients, the challenging task in this study was to create low-FODMAP cookies and crackers from flours with high FODMAP contents (wholegrain wheat and wholegrain rye). Our main goal was to create low-FODMAP products that have high nutritional value, high fibre content, and satisfactory sensory quality and thus do not differ from the standard and most consumed products of these types. In order to achieve this, the application of biotechnological tools, coupled with process optimization, was used and evaluated. The biotechnological tools included endogenous and exogenous enzymes and fermentation [3,19]. Endogenous enzymes originating from wholegrain flour were activated by means of prolonged dough resting, while the exogenous enzymes originated from chicory (in chicory root extract), wheat malt, and bakers’ yeast.

## 2. Materials and Methods

### 2.1. Materials

The wholegrain wheat flour, wholegrain rye flour, chicory root, fresh baker’s yeast (*Saccharomyces cerevisiae*), salt (NaCl), shortening (vegetable fat with a melting point of 36–38 °C, Puratos, Beograd, Serbia), sucrose, and chemical rising agents (sodium hydrogen carbonate and ammonium hydrogen carbonate) that were used in this study were acquired from a local market. The wheat cultivars Nataša and Izalco (cultivars developed by the Institute of Field and Vegetable Crops, Novi Sad, Serbia) were used for the production of wheat malt.

### 2.2. Production of Chicory Extract

The herbal extract of chicory (*Cichoriae Radix*) was produced from dried chicory roots, obtained from the Institute of Medicinal Plant Research “Dr. Josif Pančić”. The dried chicory roots were first washed with distilled water and mixed with 100 mM of sodium acetate buffer (pH 5) in a 1:10 ratio (*w*/*w*). The mixture was left for 72 h at 4 °C and subsequently filtered through cheesecloth (cotton gauze). The obtained chicory extract was frozen and kept at −20 °C before use. The chicory extract had an FEH activity of 6.5 ± 0.8 U ((μmoL fructose liberated × h^−1^)/g raw plant material), which was determined using the method proposed by Krivorotova and Sereikaite [20].

### 2.3. Production of Wheat Malt

The production of wheat malt from two domestic varieties was achieved according to the modified standard MEBAK procedure described by Krstanović et al. [21]. Micromalting (steeping and germination) was performed in a controlled-atmosphere incubator (Bluepard MGC-250 Plant Growth Chamber, Shanghai Bluepard Instruments Co., Ltd., Shanghai, China) at 14.5 °C and with a humidity of 85% to achieve a grain moisture content of 44.5%. After steeping, the grains were left to germinate once the grain moisture was adjusted to 44% (by means of spraying). Then, the produced malt was kilned at 40 °C for 24 h (Memmert IN160, Memmert GmbH+Co. KG, Schwabach, Germany), transferred into paper bags, and kept at room temperature for 30 days to stabilize their enzyme activity and moisture content. Wheat malt from the variety Nataša was added as a 30% substitution for wholegrain wheat flour, while wheat malt from the variety Izalco was added as a 5% substitution for wholegrain rye flour.

### 2.4. Cookie Preparation Procedure

The cookie samples were prepared using the procedure described in Table 1. After the mixing and resting phase, all doughs were laminated to 6 mm, and a No. 4 (diameter: 4 cm) round mould was used to shape the cookies. After baking, the cookies were left to cool and placed in plastic bags for further analysis.

### 2.5. Cracker Preparation Procedure

Cracker samples were prepared using the procedure described in Table 2. After the fermentation and resting phase, all the doughs were laminated to 6 mm, and a No. 3 (diameter: 3 cm) round mould was used to shape the crackers. After baking, the crackers were left to cool and placed in plastic bags for further analysis.

### 2.6. Quantification of FODMAPs Using HPAEC-PAD Method

All the samples (final samples after baking) were then freeze-dried and ground. A total of 50 mL of ultrapure water was added to 0.5 g of ground sample, and after that, the samples were placed in a boiling water bath for 10 min. Afterwards, the samples were cooled to room temperature and centrifuged at 8000 rpm for 10 min at 4 °C, and the separated supernatant was frozen at −20 °C (to enable the separation of any fat present, thus avoiding the use of organic solvents). The frozen supernatant was thawed and then centrifuged at a speed of 8000 rpm for 5 min at 4 °C. Finally, 5 mL of the prepared sample was transferred to vials for the HPAEC-PAD procedure.

Targeted carbohydrates were separated and quantified using a Dionex ICS-6000+ system (Sunnivale, CA, USA) and an electrochemical detector (ED) with a gold working electrode and a AgCl reference electrode.

For monosaccharides, disaccharides, and polyols, the eluents consisted of 10 mM NaOH, 200 mM NaOH, and ultrapure water. The separation of monosaccharides, disaccharides, and polyols was performed on a Thermo Scientific Dionex CarboPac PA20 analytical column (3 × 150 mm), with an appropriate protective column, using an isocratic elution according to the method of Weitzhandler et al. [22].

The separation of oligosaccharides (FOS and GOS) was carried out using a Thermo Scientific Dionex CarboPac PA200 analytical column (3 × 250 mm), with an appropriate guard column, using a gradient elution according to the method of Ispiryan et al. [23]. The eluents that were used for separation were 200 mM NaOH, 500 mM NaOH, 500 mM NaOAc, and ultrapure water. Separation was performed at a temperature of 30 °C on both columns, while detection was performed at 25 °C.

For the quantification of FODMAPs, an extrapure 50% sodium hydroxide solution (in water) (Fisher Chemical™, Bruxelles, Belgium), sodium acetate (99%+, NaOAc) (Thermo Scientific, Leipzig, Germany), and standards were used. The standards included fructose, galactose, glucose, sucrose, mannitol, sorbitol, 1-kestose, nystose, stachyose hydrate (Sigma-Aldrich, Taufkirchen, Germany), xylitol, rhamnose monohydrate, raffinose pentahydrate (Roth, Lichtentanne, Germany), lactose monohydrate, melibiose, maltitol (Thermo Scientific, Germany), verbascose, and 1,1,1-kestopentaose (Megazyme, Republic of Ireland, Wicklow, Ireland). All the carbohydrate reference standards were of >98% purity, except for 1,1,1-kestopentaose (80% purity). Ultrapure water, with a resistivity of 18.2 MΩ·cm and a total organic carbon (TOC) content of <5 ppb (ASTM Type I), was used for the preparation of the HPAEC-PAD eluents, all standard solutions, and samples; it was obtained using an Adrona Crystal-pure water purification system (Riga, Latvia).

### 2.7. Total Fructans

The total fructan content was determined using the Megazyme fructan assay kit, K-FRUC (Megazyme Ltd., Wicklow, Ireland), as a reference method.

### 2.8. Textural Properties

The biscuits’ fracture strength was measured on a TA-XTplus (Stable Micro Systems, Godalming, Surrey, UK) texturometer using a 3-point bending test. A 3-point bending rig and a 30 kg cell were used with the following test settings: pre- and post-test speeds: 1 and 10 mm/s, respectively; test speed: 3 mm/s; probe distance: 8 mm; and distance between the supports: 24 mm. The maximum force recorded at sample fracture was interpreted as the “hardness”, while the mean distance that the probe travelled until the point of fracture was the “fracturability”. A shorter probe distance indicates higher fracturability of a sample. For each sample, 3 replicates were analysed.

### 2.9. Sensory Analyses

The sensory analysis of the crackers and cookies was carried out in a sensory testing laboratory with appropriate control of the environmental conditions (under artificial daylight and temperature control (22 °C)) according to the ISO 8589:2007 standard [24]. A panel of seven highly trained and experienced assessors (3 male and 4 female), between 22 and 55 years old, who had more than two years of experience in the examination of commercial wholegrain products and products developed within scientific research projects was selected. The panel was trained over ten sessions of 2 h per day. The training included the identification of sensory descriptors (Table 3) and evaluation of the intensity of relevant sensory attributes. During descriptive sensory assessments, panellists were asked to score seven parameters, appearance, colour, porosity, aroma, taste, odour, and aftertaste, evaluating the intensity of each attribute on a 9-point scale from 9 (“extremely good”) to 1 (“extremely bad”). In total, three crackers and three cookies were evaluated in two separate sessions, 24 h after baking. All of the samples were coded with random three-digit numbers and served with water with a low mineral content to rinse the palate between individual samples. Prior to the assessment, a two-hour session was held to select relevant sensory attributes (Table 3).

### 2.10. Statistical Analyses

The experiments were performed in triplicates. Values are expressed as means ± standard deviation. The statistical calculations were carried out using MS Excel 2010.

## 3. Results and Discussion

### 3.1. FODMAP Compounds Reduction During Creation of Cookies and Crackers

Instead of avoiding FODMAP-containing food products, the FODMAP content of foods can be reduced by means of bioprocessing, such as enzymatic treatment, fermentation, or germination. During fermentation and germination, several enzymatic processes take place, leading to polymer degradation, the generation of metabolites, and other major changes. Germinated seeds or malt, which are also used as additives (as partial flour substitutes), could be considered as a valuable source of endogenous enzymes that have the potential to reduce the contents of specific FODMAPs. By using enzymatic treatment, the FODMAPs can be targeted more specifically than when using fermentation and germination [25]. In order to fully address the issue of the complexity and variety of cut-off values for each FODMAP category in terms of contents per serving and g/100 g of product “as is”, the currently available definitions from regulation authorities for the markets of Europe, USA, Canada, and Australia are summarized in Table 4 [26,27,28,29,30].

The contents of different categories of FODMAP compounds in the created cookies and crackers are presented in Table 5 and Table 6, respectively.

In regard to other literature data and the definition of low/high levels of FODMAPs, the higher values of specific FODMAP categories were used in each sample for the determination of low- or high-FODMAP status (rating based on Table 1). In addition, the presence of significant amounts of GOS in the cereal-based products was evident, which clearly affects their low–high-FODMAP status. This contrasts with previous findings that FOS are the predominant FODMAP oligosaccharides in grains and grain-based products [31].

Based on the results regarding the contents of FODMAP compounds in the cookie and cracker samples, obtained using three different processes for FODMAP content reduction, it is evident that the prolonged dough resting time after kneading (the mixing of all ingredients) only activated the endogenous enzymes of the flour and yeast efficiently in the case of yeast cracker production. The substitution of some of the flour with wheat malt in the dough proved to be a more effective procedure for reducing the FODMAP content, because with this method, an ingredient with the already activated enzymes was added into the formulation of cookies and crackers. However, the level of enzymatic activity of wheat malt significantly depends on the wheat variety. Thus, in the case of malt produced from the wheat variety Nataša, high enzymatic activity was achieved with the 30% substitution of wholegrain wheat flour, resulting in the creation of low-FODMAP cookies, while its combination with the fresh baker’s yeast enabled the production of low-FODMAP wholegrain wheat yeast crackers.

The substitution of wholegrain rye flour with 5% malt produced from the wheat variety Izalco did not result in a reduction in FODMAP content below the cut-off value for cookies with a dough resting time of 90 min. Our hypothesis was that the resting time of 90 min was not sufficient for the malt enzymes to reach their full potential, and the degradation of FODMAP compounds was insufficient. This was confirmed by the successful creation of low-FODMAP wholegrain rye cookies after the dough resting time (after kneading) was extended to 120 and 150 min.

Aqueous extract from chicory root, a natural source of inulin and enzymes for the degradation of inulin and its products, introduced a sufficient number of enzymes into the formulation of the wholegrain wheat crackers, thus resulting in the creation of a low-FODMAP product. It is intriguing to employ herbs and their extracts as food supplements due to their rising consumption. These herbs could provide potent biochemicals including antioxidants, anticarcinogens, and antimutagens [13].

The addition of fresh baker’s yeast, without additional sources of fructanases, was sufficient for the creation of low-FODMAP crackers from both wholegrain wheat and wholegrain rye flour. An understanding of baker’s yeast metabolism is a crucial aspect of the development of a FODMAP reduction strategy for crackers. *S. cerevisiae* possesses invertase [32], which has a higher affinity towards short-chain fructans and quickly degrades fructans, with a degree of polymerization (DP) of up to 5 in the first hour of fermentation. However, the degradation of fructans with higher DPs is slower [33], leading to the necessity for a prolonged dough resting time. According to Struyf et al. [34], yeast invertase can degrade fructooligosaccharides with a DP > 4 and also has the ability to degrade branched fructan structures, such as the branched graminan- and neo-type fructans that are present in wheat. During the first 60 min of fermentation, any remaining tri- and tetrasaccharides were mostly hydrolysed, and the pentasaccharide content was reduced to about 50%. Additionally, fructosyltransferase and fructose-bisphosphate aldolase, often called aldolase, contribute to the yeast’s ability to reduce the content of FOS compounds [35,36]. Furthermore, the yeast α-galactosidases is responsible for GOS reductions [37]. Although inulin is not present in wheat flour, the inulinase that is found in bakers’ yeast might be of importance for the hydrolysis of wheat fructans, since some of the cereal fructans have been reported to have an “inulin-type” structure, specifically chains with (2→I)-fructofuranosidic linkages between the fructose units [38].

### 3.2. Texture Properties of Wholegrain Crackers and Cookies

**Figure 1:**
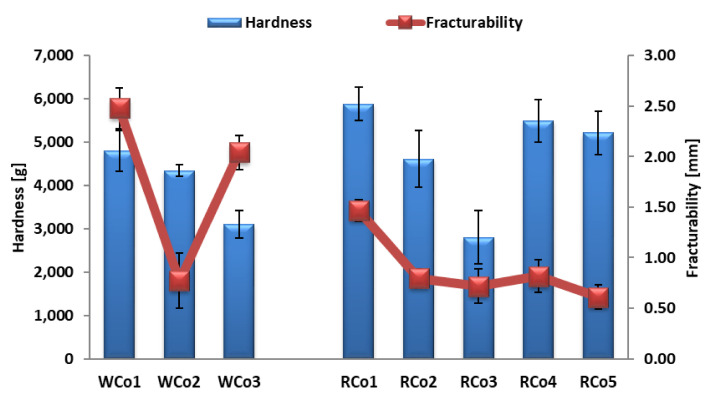
Textural properties of produced low-FODMAP wholegrain cookies.

**Figure 2:**
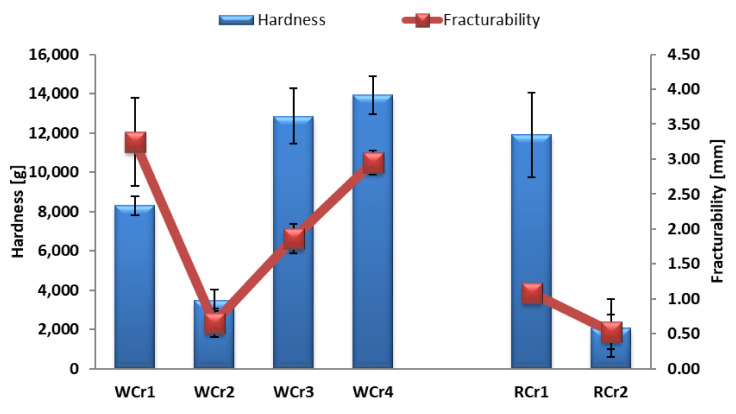
Textural properties of produced low-FODMAP wholegrain crackers.

The textural properties (hardness and fracturability) of the wholegrain wheat and wholegrain rye cookies and crackers are presented in Figure 1 and Figure 2, respectively.

The addition of dietary fibre sources into the low-FODMAP cookie formulation resulted in less hardness in comparison to the results for the high-FODMAP cookies, which was similar to the findings of Sahin et al. [39]. The same trend was observed for both the wholegrain wheat and wholegrain rye cookies. The addition of sprouted whole wheat flour also increased the hardness of the wholegrain cookies in research conducted by Jribi et al. [40].

The wholegrain wheat low-FODMAP crackers had a higher degree of hardness than the control sample (WCr1), while the opposite trend was observed for the wholegrain rye low-FODMAP crackers.

### 3.3. Sensory Profile of Cookies and Crackers

**Figure 3:**
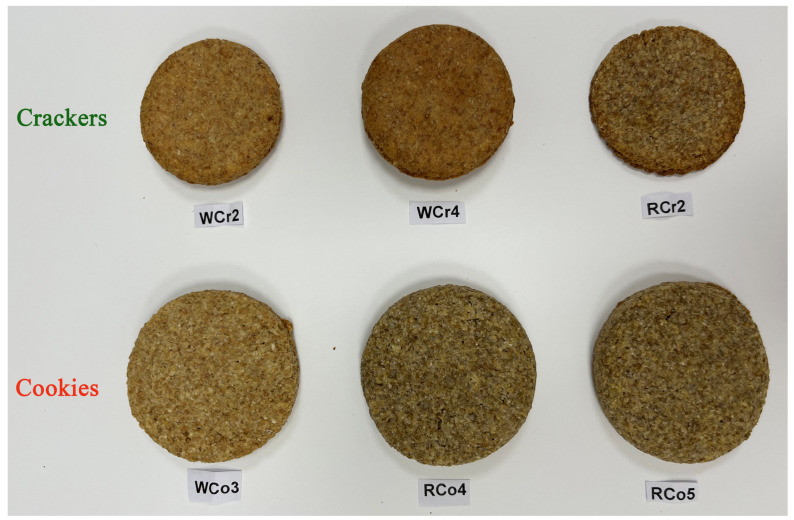
Low-FODMAP cookies and crackers.

**Figure 4:**
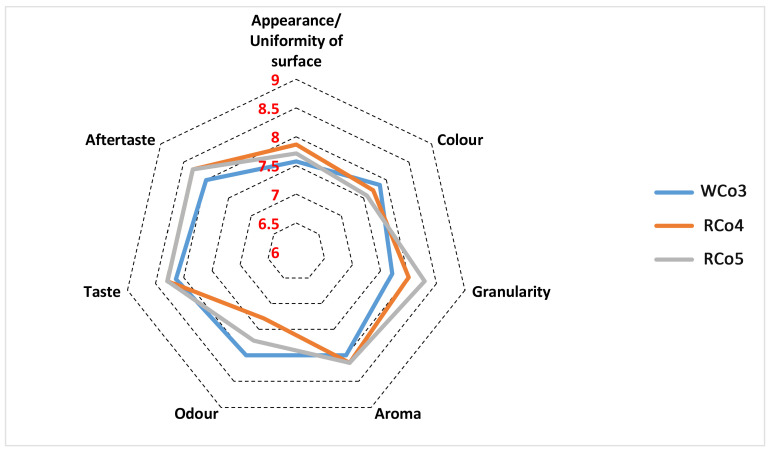
Acceptability of appearance, colour, granularity, aroma, odour, taste, and aftertaste of cookies based on descriptive sensory analysis with 9-point scale from 9 (“extremely good”) to 1 (“extremely bad”). WCo3—wholegrain wheat cookies with the addition of 30% wheat malt from the variety Nataša; RCo4—Wholegrain rye cookies with the addition of 5% wheat malt from the variety Izalco and prolonged resting time of 120 min; RCo5—Wholegrain rye cookies with the addition of 5% wheat malt from the variety Izalco and prolonged resting time of 150 min.

**Figure 5:**
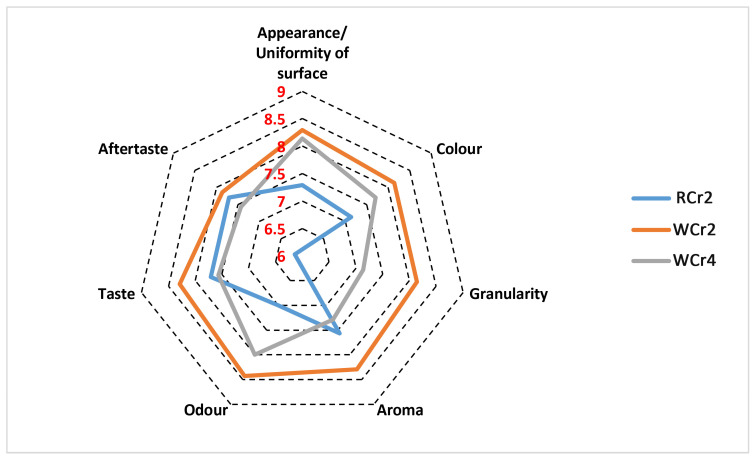
Acceptability of appearance, colour, granularity, aroma, odour, taste, and aftertaste of crackers based on descriptive sensory analysis with 9-point scale from 9 (“extremely good”) to 1 (“extremely bad”). RCr2—wholegrain rye crackers with the addition of 1.83% of fresh baker’s yeast; WCr2—Wholegrain wheat crackers with the addition of chicory extract; WCr4—Wholegrain rye cookies with the addition of 1.83% of fresh baker’s yeast.

The created low-FODMAP cookies and crackers are presented in Figure 3. The results of the descriptive sensory analyses of the three low-FODMAP cookies and three low-FODMAP crackers are shown in Figure 4 and Figure 5, respectively.

Regarding the sensory properties of the evaluated cookie samples, no significant differences were found between the samples. For all samples, their appearance, colour, and odour achieved an average value > 7, while aroma, taste, and aftertaste (perceived as a positive characteristic) had an average score > 8. The obtained results suggest that both Nataša and Izalco can be used in cookie formulations as substitutes (using 30 and 5%, respectively) for wholegrain wheat and rye flour in order to reduce FODMAPs without altering sensory properties.

There was a statistically significant difference (*p* < 0.05) between the appearance/uniformity of the surface and colour of cracker WCr2, with an average value > 8 compared with cracker RCr2, which had average values of 7.29 and 7.14. Further, the granularity of cracker WCr2 had a statistically significantly higher score, with an average value of 8.14, than cracker RCr2, which had an average value of 5.86. No significant difference was found between the crackers in terms of taste and aftertaste, which were all scored with average values > 7. The sample WCr2 had the highest average values for aroma and odour: 8.28 and 8.42, respectively. Based on the obtained results of the sensory analysis, it can be observed that the addition of chicory extract had no negative impact on the taste and aftertaste, with scored values > 8, and the sample with chicory extract had the highest average value for aroma (8.28). The addition of malt did not negatively affect either the aroma, taste, or aftertaste (perceived as a positive characteristic) of the cracker samples, which were all rated highly, with average scores > 8. The obtained results are in agreement with the findings of Ivanišová et al. [41], who recommended the application of chicory fibre in the production of biscuits to adjust the sensory properties of bakery products.

## 4. Conclusions

In the current literature, the only method for producing low-FODMAP cookies and crackers are fortification, the substitution of some of the wheat and/or rye flour with a cereal flour that is low in FODMAP compounds, or the use of commercially available fibre preparations and vital gluten as fillers to dilute the content of FODMAP compounds in wheat and rye flour. This study is the first of its kind to employ biotechnological tools (based on the exploitation of endogenous and exogenous enzymes from several plant materials and fermentation) coupled with process optimization to reduce the FODMAP content in flours with a high FODMAP content (wholegrain wheat and wholegrain rye) and create wholegrain low-FODMAP cookies and crackers. The created low-FODMAP cookies and crackers, produced using wholegrain wheat and rye, also had excellent sensory properties, and the addition of aqueous chicory extract and wheat malts had a positive effect on their overall appeal. Aside from the chicory extract and wheat malts, the baker’s yeast and prolonged dough resting time had an exceptional effect on the reduction in FODMAP content, with a moderate influence on the sensory properties and neutral (passable) overall appeal.

We also clearly identified significant amounts of GOS in the cereal-based products, in contrast to previous findings that FOS are the predominant FODMAP oligosaccharide in grains and grain-based products. In addition, the inclusion of melibiose and verbascose to the total GOS content provided better insight into the total FODMAP content and thus enhanced the definition of low- and high-FODMAP foods. In terms of the oligosaccharide fraction of the compounds classified as FODMAPs, and taking into account the significant content of GOS (identified in the analysed samples) and differences in FOS and total fructans as FODMAP constituents, the general conclusion is that in order to define high-FODMAP foods, the most reliable criterion is the content of total oligosaccharides (the sum of FOS and GOS).

In order to bring the developed low-FODMAP cookies and crackers closer to commercialization, further development will focus on the analysis of their shelf life its extension, conducting consumer acceptance tests on a large scale (above 300 participants and, if possible, in several countries), and the determination of their biological activity.

## Figures and Tables

**Table 1:** Procedures for wholegrain cookie preparation.

COOKIES									
		Wholegrain Wheat			Wholegrain Rye				
	% Relative to Amount of Flour	WCo1	WCo2	WCo3	RCo1	RCo2	RCo3	RCo4	RCo5
1.	Wholegrain wheat flour	100	100	70	0	0	0	0	0
2.	Wholegrain rye flour	0	0	0	100	100	95	95	95
3.	Malt	0	0	30 *	0	0	5 *	5 *	5
4.	Water	31.2	31.2	31.2	20	20	20	20	20
5.	Sucrose	20	20	20	28	28	28	28	28
6.	Salt	1	1	1	1.1	1.1	1.1	1.1	1.1
7.	Shortening	20	20	20	32	32	32	32	32
8.	Sodium hydrogen carbonate	0.5	0.5	0.5	0.4	0.4	0.4	0.4	0.4
9.	Ammonium hydrogen carbonate	0.5	0.5	0.5	0.2	0.2	0.2	0.2	0.2
Conditions									
1.	Mixing 1 (min)	5	5	5	5	5	5	5	5
2.	Resting 1 (min)	20	90	90	20	90	90	120	150
3.	Mixing 2 (min)	0	5	5	0	5	5	5	5
4.	Resting 2 (min)	0	20	20	0	20	20	20	20
5.	T (°C)	30	30	30	30	30	30	30	30
6.	T baking (°C)	180	180	180	180	180	180	180	180
7.	Time of baking (min)	15	15	15	15	15	15	15	15

* Wheat malt from the variety Nataša was added as a 30% substitution for wholegrain wheat flour, while wheat malt from the variety Izalco was added as a 5% substitution for wholegrain rye flour.

**Table 2:** Procedures for wholegrain cracker preparation.

CRACKERS							
		Wholegrain Wheat				Wholegrain Rye	
	% Relative to Amount of Flour	WCr1	WCr2	WCr3	WCr4	RCr1	RCr2
1.	Wholegrain wheat flour	100	100	100	70	0	0
2.	Wholegrain rye flour	0	0	0	0	100	100
3.	Malt	0	0	0	30 *	0	0
4.	Water	51.2	0	51.2	51.2	53.6	53.6
5.	Extract	0	51.2	0	0	0	0
6.	Sucrose	5	5	5	5	0	0
7.	Salt	1.5	1.5	1.42	1.42	1.6	1.42
8.	Shortening	11	11	12.5	12.5	10	12.5
9.	Sodium bicarbonate	0.55	0.55	0.25	0.25	0.55	0.25
10.	Ammonium bicarbonate	2.5	2.5	0	0	0	0
11.	Fresh baker’s yeast	0	0	1.83	1.83	0	1.83
Conditions							
1.	Mixing 1 (min)	5	5	5	5	5	5
2.	Resting 1 (min)	20	90	180	90	20	180
3.	Mixing 2 (min)	5	5	1	5	5	1
4.	Resting 2 (min)	0	20	20	180	0	20
5.	Mixing 3 (min)	0	0	0	1	0	0
6.	Resting 3 (min)	0	0	0	20	0	0
7.	T resting/fermentation (°C)	30	40	30	30	30	30
8.	T baking (°C)	220	220	220	220	220	220
9.	Time of baking (min)	13	13	13	13	13	13

* Wheat malt from the variety Nataša was added as a 30% substitution for wholegrain wheat flour.

**Table 3:** Sensory descriptors and their definition.

Sensory Attribute	Description
Appearance/Uniformity of surface	Uniform–non-uniform/smooth–rough
Colour	Degree of brownness, ranging from light brown to dark brown
Granularity	Sense of particle size and shape (larger particles–higher granularity)
Aroma	Olfactory perceptions caused by volatile substances released from a product in the mouth via the posterior nares
Odour	Overall intensity of odour (volatiles are sniffed through nose)
Taste	Overall intensity of taste (gustatory perceptions caused by soluble substances in mouth)
Aftertaste	Gustatory–olfactory sensation left in mouth after sample is swallowed or spat out (persistence of flavour after the stimulating agent has gone), perceived as a positive characteristic

**Table 4:** Serving sizes and cut-off values for FODMAP compounds per serving and per g/100 g “as is” for cookies and crackers.

		**COOKIES**	**CRACKERS**
Serving size		30 g	30 g
FODMAP		**COOKIES**	**CRACKERS**
Cut-off values	g per serving	g/100 g “as is”	
Fructose in excess of glucose	<0.15	<0.5	<0.5
Total oligosaccharides (fructans and galactooligosaccharides)	<0.3	<1	<1
Total polyoles	<0.4 (or <0.2 mannitol or sorbitol)	1.33 (0.67)	1.33 (0.67)
Lactose	<1	<3.33	<3.33

**Table 5:** Contents of different categories of FODMAP compounds in cookies (g/100 g “as is”) *.

	Wholegrain Wheat COOKIES			Wholegrain Rye COOKIES				
	WCo1	WCo2	WCo3	RCo1	RCo2	RCo3	RCo4	RCo5
Excess fructose	0	0	0	0	0	0	0	0
Lactose	0	0	0	0	0	0	0	0
Sorbitol	0	0	0	0.004	0	0.012	0	0
Mannitol	0	0	0	0	0	0.053	0	0
Total polyols	0.006	0.01	0.011	0.02	0.009	0.087	0.019	0.061
FOS	0.461	0.574	0.225	0.615	0.647	1.169	0.132	0.089
GOS	0.179	0.111	0.106	0.159	0.094	0.24	0.069	0.061
GOS+melibiose	0.18	0.122	0.122	0.16	0.096	0.268	0.07	0.07
Total oligosaccharides	0.642	0.696	0.347	0.776	0.743	1.437	0.202	0.159
FODMAP rating *	Low	Low	Low	Low	Low	High	Low	Low
Total fructans	1.739	1.602	0.768	2.673	2.592	1.547	1.063	0.805
FODMAP rating *	High	High	Low	High	High	High	High	Low

* Standard deviation of analysed samples was in range of 4.1 to 5.8%; FODMAP rating based on cut-off values from Table 4.

**Table 6:** Contents of different categories of FODMAP compounds in crackers (g/100 g “as is”) *.

	Wholegrain Wheat CRACKERS				Wholegrain Rye CRACKERS	
	WCr1	WCr2	WCr3	WCr4	RCr1	RCr2
Excess fructose	0	0	0.083	0.008	0	0.015
Lactose	0	0	0	0	0	0
Sorbitol	0	0	0	0	0	0
Mannitol	0	0	0.014	0.007	0	0
Total polyols	0	0	0.062	0.123	0.014	0.152
FOS	0.868	0.523	0.563	0.432	0.816	0.138
GOS	0.268	0.093	0	0	0.292	0
GOS+melibiose	0.272	0.148	0.121	0.032	0.296	0.044
Total oligosaccharides	1.14	0.671	0.684	0.464	1.112	0.182
FODMAP rating *	High	Low	Low	Low	High	Low
Total fructans	1.46	1.631	0.565	0.437	2.202	0.932
FODMAP rating *	High	High	Low	Low	High	Low

* Standard deviation of analysed samples was in range of 4.2 to 5.6%; FODMAP rating based on cut-off values from Table 4.

## Data Availability

The original contributions presented in the study are included in the article; further inquiries can be directed to the corresponding author.
